# Telemedicine in Oral and Maxillofacial Surgery: A Narrative Review of Clinical Applications, Outcomes and Future Directions

**DOI:** 10.3390/jcm15020452

**Published:** 2026-01-07

**Authors:** Luigi Angelo Vaira, Valentina Micheluzzi, Jerome R. Lechien, Antonino Maniaci, Fabio Maglitto, Giovanni Cammaroto, Stefania Troise, Carlos M. Chiesa-Estomba, Giuseppe Consorti, Giulio Cirignaco, Alberto Maria Saibene, Giannicola Iannella, Carlos Navarro-Cuéllar, Giovanni Maria Soro, Giovanni Salzano, Gavino Casu, Giacomo De Riu

**Affiliations:** 1 Maxillofacial Surgery Operative Unit, Department of Medicine, Surgery and Pharmacy, University of Sassari, 07100 Sassari, Italy; gderiu@uniss.it; 2 Clinical and Interventional Cardiology, Sassari University Hospital, 07100 Sassari, Italy; valentina.micheluzzi@aouss.it (V.M.); gcasu2@uniss.it (G.C.); 3 Department of Surgery, Mons School of Medicine, UMONS, Research Institute for Health Sciences and Technology, University of Mons (UMons), 7000 Mons, Belgium; jerome.lechien@umons.ac.be; 4 Department of Otolaryngology-Head Neck Surgery, Elsan Polyclinic of Poitiers, 86000 Poitiers, France; 5 Department of Medicine and Surgery, University of Enna Kore, 94019 Enna, Italy; tnmaniaci29@gmail.com; 6 Head and Neck Section, Department of Neurosciences, Reproductive and Odontostomatological Science, Federico II University of Naples, 80131 Naples, Italy; fmaglitto@gmail.com (F.M.); stefy.troise@gmail.com (S.T.); giovannisalzanomd@gmail.com (G.S.); 7 Head and Neck Department, ENT & Oral Surgery Unity, G.B. Morgagni, L. Pierantoni Hospital, 47121 Forlì, Italy; giovanni.cammaroto@hotmail.com; 8 Department of Otorhinolaryngology-Head & Neck Surgery, Hospital Universitario Donostia, 20001 San Sebastian, Spain; chiesaestomba86@gmail.com; 9 Division of Maxillofacial Surgery, Department of Neurological Sciences, Marche University Hospitals—Umberto I, 60129 Ancona, Italy; giuseppe.consorti@ospedaliriuniti.marche.it (G.C.); giuliocirignaco@gmail.com (G.C.); 10 Department of Biomedical Sciences and Public Health, Polytechnic University of Marche, 60129 Ancona, Italy; 11 Otolaryngology Unit, Department of Health Sciences, Santi Paolo e Carlo Hospital, University of Milan, 81841 Milan, Italy; alberto.saibene@gmail.com; 12 Department of ‘Organi di Senso’, University “Sapienza”, Viale dell’Università 33, 00185 Rome, Italy; giannicola.iannella@uniroma1.it; 13 Maxillofacial Surgery Department, Hospital Gregorio Marañon, Universidad Complutense de Madrid, 28040 Madrid, Spain; cnavarrocuellar@gmail.com; 14 General Management Office, University of Sassari, 07100 Sassari, Italy; gmsoro@uniss.it

**Keywords:** telemedicine, telehealth, oral and maxillofacial surgery, teleradiology, postoperative care, trauma triage

## Abstract

**Objectives:** Telemedicine has rapidly expanded in oral and maxillofacial surgery (OMFS), especially during the COVID-19 pandemic, but its specific roles and limitations across the care pathway remain unclear. This narrative review aimed to map telemedicine modalities and indications in OMFS, summarize reported outcomes, and identify priorities for future research. **Methods:** A narrative synthesis was undertaken after a systematic search of medical and engineering databases to 10 October 2025. Studies applying telemedicine, telehealth, telepresence or teleradiology to OMFS practice were eligible, including trials, observational cohorts, technical reports and surveys. Data were extracted in duplicate and organized thematically; heterogeneity precluded meta-analysis. **Results:** Fifty studies met the inclusion criteria. Telemedicine was mainly used for preoperative consultation and triage, postoperative follow-up, trauma teleradiology and tele-expertise, oncologic and oral medicine follow-up, temporomandibular disorders, and education or humanitarian work. In low-risk outpatient and postoperative settings, remote consultations showed high concordance with in-person plans, similar complication or reattendance rates, reduced travel, and high satisfaction. In trauma networks, telemedicine supported timely triage and reduced unnecessary inter-hospital transfers. Evidence in oral oncology and complex mucosal disease was more cautious, favouring hybrid models and escalation to face-to-face assessment. Data on cost-effectiveness and impacts on equity were limited. **Conclusions:** Telemedicine in OMFS has moved from niche innovation to a pragmatic adjunct across the clinical pathway. Current evidence supports its use for selected pre- and postoperative care and trauma triage within risk-stratified hybrid models, while underscoring the need for stronger comparative and implementation studies, clear governance on equity and data protection, and alignment with wider digital and AI-enabled health systems.

## 1. Introduction

Telemedicine has shifted from a pandemic-driven workaround to a durable component of oral and maxillofacial surgery (OMFS) workflows, spanning triage, consultation, postoperative follow-up, education, and humanitarian collaboration. Early in COVID-19, expert guidance encouraged rapid adoption alongside infection-control, screening, and redesigned patient pathways to preserve access while minimizing exposure risks [1]. Subsequent utilization studies show telehealth stabilized as a complementary modality: by 2022, most new OMFS patients had returned to in-person visits, whereas return visits continued to leverage remote platforms at meaningful rates, reflecting a pragmatic “hybrid” equilibrium [2,3,4]. Patient and clinician surveys during and after the acute pandemic phase consistently report high satisfaction and willingness to continue remote models—particularly for selected indications—while underscoring persistent concerns about examination limitations, image quality, privacy, and fit within clinical pathways [4,5,6,7].

Across indications, the evidence is heterogenous but directionally consistent. For preoperative assessment and dentoalveolar surgery, telemedicine has repeatedly demonstrated high plan concordance and operational efficiency, enabling most patients to proceed to treatment without additional preoperative work-up [8]. A randomized comparison of postoperative reviews after third-molar surgery found similar satisfaction to in-person care and higher perceived cost-effectiveness for telemedicine, supporting substitution in low-risk contexts [9]. In trauma, teleradiology supports appropriate triage from peripheral hospitals, reducing unnecessary transfers and focusing limited specialist capacity on true surgical candidates [10,11]. For oncology, hybrid models have supported follow-up communication and risk-stratified surveillance, while recent work explores asynchronous remote imaging from community dental clinics to flag early oral cancers—an area with high potential but still limited accuracy data beyond pilot series [3,12,13,14,15,16,17,18,19,20,21,22,23,24]. At the same time, specialties with heavy reliance on nuanced visual–tactile examination (e.g., Oral Medicine) warn that telephone/video alone can be insufficient for first presentations or complex mucosal disease, advocating careful triage and clear escalation thresholds [4,15].

Beyond clinical outcomes, system and policy dimensions shape adoption. Financial analyses suggest near-parity reimbursement between telemedicine and in-person OMFS visits in an academic practice, with only small differences in reimbursement-to-charge ratios—data that may reassure clinics considering sustained hybrid services [16]. Consultant surveys in Australia indicate strong willingness to continue telehealth contingent on addressing diagnostic uncertainty, postoperative monitoring limitations, and the need to protect vulnerable patient groups—all themes that point to the importance of standardized imaging, secure platforms, and co-designed protocols [5]. In Latin America and the Caribbean, experts identify structural barriers to early oral cancer diagnosis—unclear referral pathways, low reporting, and workforce constraints—while strongly endorsing online education and telemedicine as pragmatic levers to narrow gaps [17]. Collectively, these findings argue that equity gains are possible, but not guaranteed, without intentional design to overcome the digital divide and pathway fragmentation.

The OMFS community has also advanced tele-education and remote collaboration. Tele-OSCEs achieved comparable performance to prior in-person examinations for history-taking/consultation competencies (with predictable limits for practical skills) [19], and department-level protocols (e.g., the SEF framework) demonstrated reliable, high-quality telementoring and multi-stream video for surgical education [20]. In humanitarian contexts, pre-mission teleconsultation and postoperative tele-follow-up improved case selection, resource planning, and continuity of care across borders; mission reports during and after the pandemic emphasize how online tools can mitigate logistical constraints in resource-limited settings [21,22].

From a methods perspective, the pre-COVID evidence base comprised small observational series, diagnostic studies, and early service reports; pandemic-era publications expanded the volume but not uniformly the rigor. A 2019 scoping review mapped 34 patient-centered eHealth interventions in OMFS and highlighted that most work remained in development/feasibility phases, with no implementation studies at the time [18]. Post-2020, the field gained service evaluations, cohort studies, mixed-methods surveys, and at least one randomized comparison in a well-defined postoperative setting [9], yet high-quality comparative trials and cost-effectiveness analyses remain sparse. Moreover, consistent technical standards for remote intraoral imaging, lighting and view protocols, device specifications, and integration with electronic records are unevenly reported, complicating synthesis and scale-up. Ethical–legal considerations (consent, data security, cross-jurisdictional care) are frequently acknowledged but rarely operationalized in study designs [3,6].

This review aims to: (i) classify telemedicine modalities used in OMFS (synchronous video/telephone, asynchronous store-and-forward, teleradiology, app-based monitoring, hybrid pathways); (ii) map their clinical indications and settings (oncology, trauma, temporomandibular disorders, dentoalveolar surgery, orthodontics, head-and-neck follow-up, education, and humanitarian missions); (iii) synthesize reported outcomes (diagnostic/plan accuracy, operational efficiency, satisfaction, economics, safety, equity); (iv) identify barriers/facilitators and policy levers (reimbursement, privacy, standards, workflow integration); and (v) delineate evidence gaps and priority directions (standardized imaging protocols, implementation science, rigorous comparative trials, and the role of AI-enabled image quality assurance and decision support).

## 2. Materials and Methods

This review was conceived as a narrative synthesis informed by a structured, multi-database search rather than as a formal systematic review. A question-driven approach was adopted to map how telemedicine has been applied across OMFS, recognizing the heterogeneity of available evidence, which includes service evaluations, observational cohorts, randomized trials, technical descriptions and expert commentaries. The conduct and reporting of the review were guided by general principles for transparent narrative and scoping reviews, with explicit description of information sources, eligibility criteria, study selection and data extraction, while formal protocol registration and risk-of-bias scoring were not undertaken because of the diversity of study types and outcomes [25,26].

Although methodological elements frequently adopted in scoping reviews (e.g., comprehensive multi-database searching, duplicate screening, and structured charting) were implemented, the review was framed as a narrative synthesis because the primary aim was an interpretive, clinically oriented integration of applications, outcomes, and implementation considerations in OMFS, rather than a formal evidence-mapping exercise conducted and reported according to PRISMA-ScR guidance [25,26]. Furthermore, the marked heterogeneity in indications, telemedicine modalities, comparators, and outcome reporting across the included studies was considered to limit the feasibility of standardized critical appraisal and of direct comparative conclusions typical of systematic reviews. Consequently, a thematic narrative synthesis approach was adopted, while structured search and selection procedures were retained to enhance transparency and minimize selection bias [26].

### 2.1. Search Strategy and Information Sources

A comprehensive electronic search was carried out in PubMed/MEDLINE (National Library of Medicine, Bethesda, MD, USA), Embase (Elsevier B.V., Amsterdam, The Netherlands), Scopus (Elsevier B.V., Amsterdam, The Netherlands), Web of Science Core Collection (Clarivate Analytics, Philadelphia, PA, USA), Cochrane Library (Cochrane, London, UK) (Cochrane Central Register of Controlled Trials and Cochrane Reviews), and IEEE Xplore (IEEE, Piscataway, NJ, USA). All database searches were completed on 10 October 2025.

For each database, combinations of controlled vocabulary (e.g., MeSH, Emtree) and free-text terms related to telemedicine and OMFS were employed. Full search strings for each database, including database-specific syntax and filters, will be reported in Supplementary Tables S1.

In addition to database searches, reference lists of key primary articles and existing overviews of eHealth and telemedicine in OMFS were examined to identify additional eligible studies that might not have been retrieved by keyword searches alone. Citation tracking of influential early telemedicine reports in OMFS was also undertaken to detect later follow-up or implementation studies.

### 2.2. Eligibility Criteria and Study Selection

Studies were considered eligible if the use of telemedicine, telehealth or closely related digital remote-care tools was reported in settings directly relevant to OMFS. Eligible contexts included clinical care within recognized OMFS domains (i.e., dentoalveolar surgery, maxillofacial trauma, temporomandibular disorders, orthognathic and reconstructive surgery, oncologic and head-and-neck follow-up, oral medicine and oral oncology), organizational models and service redesign for OMFS departments where telemedicine played a central role, and education, training or humanitarian missions in which remote technologies were applied specifically to OMFS practice.

Studies focused exclusively on general teledentistry without a clear interface with OMFS pathways, purely technical telecommunications reports without clinical application, non-human studies, conference abstracts without full text and non-English publications were excluded.

Title-and-abstract screening was performed independently by two reviewers with training in OMFS and clinical research methodology. Each record was assessed against the predefined eligibility criteria. Full-text articles were obtained for all records judged potentially relevant by either reviewer. Full-text screening and final inclusion decisions were also carried out in duplicate. Disagreements at either stage were resolved by discussion; when consensus could not be reached, a third senior reviewer adjudicated. Reasons for exclusion at full-text stage (for example: not OMFS-specific, no actual telemedicine implementation, non-clinical technical paper) were recorded in a screening log.

Studies in oral oncology or oral medicine were retained when an OMFS team was clearly involved in diagnosis, treatment or follow-up, or when the care pathway was typical of an OMFS service. Where critical information on design, setting or intervention remained unclear even after full-text review, the study was described conservatively and was not over-interpreted in the synthesis.

For conceptual clarity, terminology was used as follows. Telemedicine was used to denote the provision of clinical services at a distance (e.g., remote consultation, triage, follow-up) using telecommunications technologies. Telehealth was treated as the broader umbrella term that may also include non-clinical activities such as education, training, and administrative support, in addition to clinical care. Teledentistry was used when the remote-care model was described within dental services and referral pathways, including interfaces with OMFS. eHealth was used to describe wider digital-health interventions (e.g., patient-centred digital tools, platforms, and systems integrating remote care with digital workflows). Because primary studies variably apply these labels, the synthesis primarily refers to ‘telemedicine’ as an umbrella term for OMFS remote-care delivery unless a specific modality or domain is being discussed [1,18,27].

### 2.3. Data Extraction and Synthesis

Data extraction was carried out using standardized evidence tables developed for this review. Extraction was performed by one reviewer and independently checked by a second reviewer; discrepancies were discussed and resolved by consensus, with arbitration by the third reviewer when necessary. For each included study, the following characteristics were recorded where reported: year of publication; country or region; clinical setting (i.e., tertiary university hospital, regional hub-and-spoke network, community practice, humanitarian mission); study design; telemedicine modality (i.e., synchronous video consultation, telephone consultation, asynchronous store-and-forward imaging, teleradiology, mobile or web-based applications, tele-education platforms); target indication or use case (i.e., trauma triage, preoperative assessment, postoperative follow-up, oncology surveillance, early oral cancer detection, temporomandibular joint disorder management, outpatient review, training or mission planning); sample size or number of telemedicine encounters; and main reported outcomes.

Outcomes were grouped into broad domains. Clinical outcomes included diagnostic accuracy or concordance of treatment plans between telemedicine and in-person assessments, appropriateness of triage, complication rates and, where available, oncologic or functional endpoints. Operational outcomes encompassed waiting times and time to treatment, numbers of physical transfers or face-to-face visits avoided, cancellation rates and measures of workflow feasibility or service continuity. Patient-reported and clinician-reported outcomes included satisfaction, perceived usefulness, perceived cost-effectiveness and willingness to continue using telemedicine. Economic outcomes were captured where available, including reimbursement-to-charge ratios, estimated travel and time savings and cost estimates associated with telemedicine implementation. When abstracts or full texts did not provide specific numerical values for an outcome, no attempt was made to infer or impute data, and only qualitative statements explicitly reported by the authors were retained.

For the narrative synthesis, studies were organized into thematic clusters reflecting both clinical domain and telemedicine function. Clusters included preoperative assessment and routine outpatient care, postoperative follow-up and monitoring (including app-based approaches), trauma and emergency care with a focus on teleradiology and tele-expertise, oncology and oral cancer (follow-up and early detection), oral medicine and mucosal disease, education and training (including tele-OSCEs and telementoring) and humanitarian or equity-focused applications. Within these clusters, distinctions were drawn between synchronous and asynchronous models and between telemedicine used as an adjunct to, versus a replacement for, in-person consultations. In view of substantial methodological and outcome heterogeneity, no formal meta-analysis was undertaken, and no pooled effect measures were calculated.

### 2.4. Considerations on Quality and Limitations

Because of the mixture of randomized trials, observational studies, surveys and descriptive reports, formal risk-of-bias tools and grading frameworks were not applied uniformly. Instead, attention was directed toward study design, sample size, clarity of outcome definitions and the degree to which authors’ conclusions were supported by their data. Pilot and feasibility studies, small case series and expert commentaries were explicitly identified as such in the synthesis, and their limitations were highlighted in the discussion. The chosen methodology was therefore intended to provide a transparent and structured overview of telemedicine in OMFS, identifying recurring patterns of benefit, common challenges and prominent evidence gaps, rather than to support definitive comparative effectiveness or cost-effectiveness conclusions.

## 3. Results

**Figure 1:**
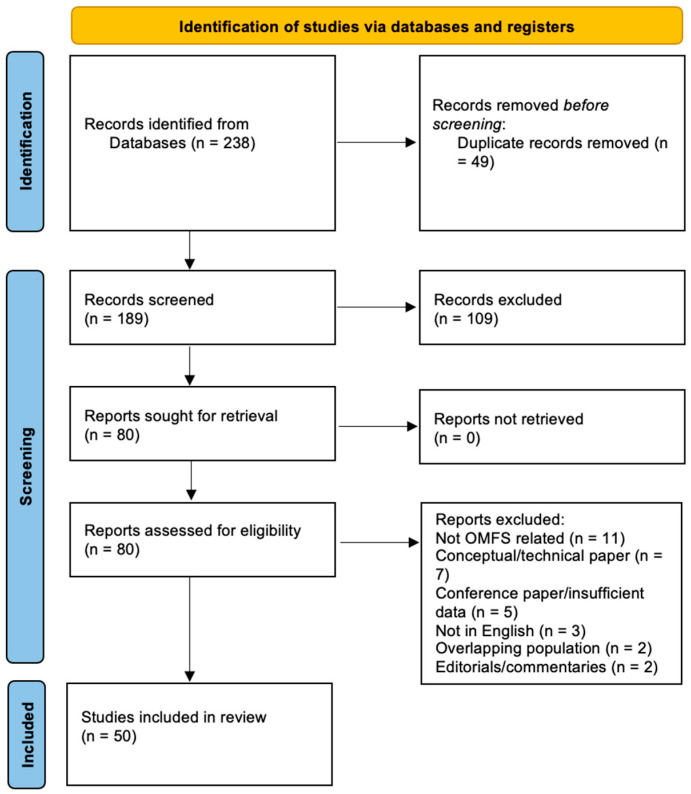
PRISMA flow diagram of the study selection process.

A total of 238 records were identified across all databases. After removal of 49 duplicates, 189 titles and abstracts were screened, leading to the exclusion of 109 records that were not simultaneously related to telemedicine and oral and maxillofacial surgery or did not involve an implemented remote-care model. Eighty full-text articles were assessed for eligibility; 30 were excluded (most commonly because the focus was general teledentistry without a clear OMFS interface, the report was a conference abstract only, or telemedicine was mentioned conceptually without primary data), leaving 50 studies for inclusion in the qualitative synthesis [1,2,3,4,5,6,7,8,9,10,11,12,13,14,15,16,17,18,19,20,21,22,23,24,27,28,29,30,31,32,33,34,35,36,37,38,39,40,41,42,43,44,45,46,47,48,49,50,51,52] (Figure 1).

These comprised randomized and non-randomized comparative studies, retrospective and prospective cohorts, diagnostic accuracy studies, case series, cross-sectional surveys, mixed-methods studies, narrative reviews and technical or organizational reports. Publication years ranged from 1999 to 2025, with a clear increase in volume from 2010 onward and a marked surge during and after the COVID-19 pandemic. Most reports originated from Europe, North America and Australia, with additional contributions from Latin America, Africa and Asia [2,5,10,17,18,20,21,22,29,31,34,39]. Tertiary university hospitals and regional hub-and-spoke networks were the most frequent settings, alongside emergency departments, dental hospitals, community practices and humanitarian missions [1,2,10,14,20,21,22,29,31,34,41,47,48,49,50] (Table 1 and Supplementary Table S2).

### 3.1. Study Designs and Telemedicine Modalities

Considerable heterogeneity in study design was observed. Comparative or cohort designs were used to evaluate diagnostic accuracy, workflow performance or patient satisfaction for telemedicine versus in-person care [8,9,10,11,12,18,28,36,37,40,43,44,48,49,50,51,52]. Surveys and mixed-methods studies explored clinician and patient perceptions, barriers and facilitators, and training or organizational impacts [3,4,5,6,7,11,16,18,19,23,27,28,31,32,33,35,38,49,51]. Narrative and scoping reviews synthesized broader eHealth and telemedicine experience in OMFS and related specialties [1,6,18,20,27,32,45]. Overall, controlled comparative evidence accounted for only a subset of the included literature, whereas a substantial proportion consisted of observational service evaluations, surveys, and descriptive feasibility reports; therefore, comparative inferences were primarily derived from studies with an explicit comparator, while non-comparative designs were used to characterize implementation, acceptability, and workflow considerations.

Across the 50 studies, telemedicine was implemented through several main modalities. Synchronous approaches included telephone clinics [3,5,6,7,11,15,19,24,28,30,40], real-time video consultations and videoconferencing, often integrated with image sharing and electronic records [1,2,10,12,13,14,18,20,21,22,29,31,34,41,45,46,47,48,49,50]. Asynchronous (store-and-forward) models were used for transmission of clinical photographs, radiographic imaging and structured clinical data, particularly in trauma triage, temporomandibular joint (TMJ) care and oncology [10,11,12,17,20,21,23,28,34,36,37,39,40,41,43,44,47,48,49,52]. Mobile applications and smartphone-based solutions were described both as communication tools and as structured platforms for remote follow-up or teleassistance [20,21,29,31,35,38,46]. A number of reports described hybrid systems, combining synchronous and asynchronous elements within integrated networks or organizational models [10,13,14,20,22,24,34,41,47,48,49,50].

### 3.2. Clinical Domains and Use Cases

Telemedicine in OMFS was applied across multiple clinical domains. For preoperative assessment and routine outpatient care, telephone or video consultations were used to triage dentoalveolar procedures, assess medical risk, define treatment plans and determine the appropriate setting (clinic vs. operating room) [1,2,3,7,8,9,11,12,14,18,19,22,24,32,36,37,40,41,42,44,50,52]. High levels of concordance between telemedicine-based and in-person surgical and anaesthetic plans were reported for dentoalveolar surgery and other routine OMFS procedures, with treatment being delivered as planned in the vast majority of cases [8,36,37,41,44,50,52]. In several cohorts and service evaluations, teleconsultations allowed many review or low-complexity visits to be managed without a subsequent face-to-face appointment [2,3,7,9,11,12,18,19,24,28,30].

For postoperative care and monitoring, telemedicine was primarily used after third molar surgery and other ambulatory procedures. Comparable patient satisfaction was reported between in-person and remote postoperative visits, with telemedicine perceived as more convenient and cost-effective by many patients [9,11,18,20,28,29,36]. A dedicated mobile health application (ExoDont) was developed to support adherence to postoperative instructions and medication after dental extractions, with favourable usability and perceived impact scores [29].

In trauma and emergency care, teleradiology and tele-expertise networks were used to triage maxillofacial fractures and soft-tissue injuries from peripheral hospitals to specialist centres [10,11,28,34,39,42,45,48,49,50]. Remote review of CT and plain radiographs allowed accurate identification of surgically relevant fractures in most studies and substantially reduced unnecessary transfers and face-to-face assessments [10,11,28,34,42,45,48,49,50]. Early work also showed that diagnostic performance by OMFS specialists using transmitted images was broadly comparable to emergency physicians using conventional radiographs, although some fracture sites (e.g., frontozygomatic and infraorbital rim) remained challenging [11,47].

In oncology and oral cancer, telemedicine was used for both follow-up and early detection. Teleconsultation models for oncologic follow-up and oral potentially malignant disorders were reported during and after COVID-19, often as part of hybrid pathways combining remote and in-person visits according to risk [1,12,13,14,17,20,22,24,30]. A remote imaging consultation system enabled early detection of several cases of oral squamous cell carcinoma referred from local dental practices, with good user satisfaction among referring dentists [23]. Surveys from Latin America and the Caribbean highlighted telemedicine and online education as widely endorsed tools to overcome structural barriers to early diagnosis and management of oral cancer [17].

Temporomandibular joint disorders were managed in one multicentre study via a store-and-forward telemedicine system linking primary care and a hospital-based OMFS unit. Most patients were diagnosed and treated conservatively in primary care with specialist guidance, with shorter delays to treatment and fewer unnecessary hospital visits [43].

Telemedicine was also used in oral medicine and mucosal disease, where clinicians reported high satisfaction with aspects such as history-taking and counselling but emphasized the limitations of remote assessment for subtle mucosal lesions and complex multisystem conditions, prompting a cautious and selective use of virtual clinics in this domain [7,15,37,40].

### 3.3. Education, Training and Organizational Models

Several studies focused on educational and organizational applications. A Tele-OSCE format in OMFS was implemented and compared with prior in-person OSCEs, with similar student performance but acknowledged limitations in practical skills assessment [19]. Residency training and didactics were widely reorganized to virtual platforms during the pandemic, with significant shifts in case exposure, teaching formats and wellness initiatives reported by program directors [1,23]. Departmental and network-level reorganization around telemedicine was described in detail in Italian and Australian settings, where teleconsultations, virtual clinics and telesemeiology were embedded into clinical workflows to maintain access while reducing infection risk [1,6,13,14,20,32,45,47,48,49,50].

Telemedicine and telepresence were also leveraged for distance telementoring, webinars and live surgical teaching, with bespoke low-cost architectures such as the SEF (Smart videosurgery, Easy teleteaching, Fast teleassistance) protocol enabling simultaneous transmission of multiple video streams to students and remote experts [20,27,31,35,38]. Earlier technical and conceptual work laid the foundations for interactive teleconsultation, navigation-assisted telesurgery and mobile video-streaming of arthroscopic and intraoperative views in craniomaxillofacial surgery [27,31,35,38,41,45,46,47].

### 3.4. Humanitarian, Equity and System-Level Perspectives

A distinct subset of studies addressed humanitarian missions and resource-limited settings. Telemedicine and online collaboration were used to plan and follow up complex maxillofacial cases in Angola and Mali, to coordinate visiting missions and to support local surgeons between visits [21,22,31]. In rural and underserved areas, telehealth was shown to be feasible and acceptable as a means to expand access to specialist dental and OMFS care, with particular relevance for populations with high travel burdens and limited local expertise [10,20,21,28,31,32,35,39].

Finally, several surveys and observational studies examined attitudes, barriers and economic aspects. High levels of clinician and patient satisfaction with remote consultations were consistently reported, especially for review visits, retainer checks, benign conditions and routine postoperative care, although concerns persisted regarding diagnostic certainty, patient confidentiality and medico-legal frameworks [2,3,4,5,6,7,9,11,15,16,18,19,27,28,32,33,37,40,49,51]. Financial analyses suggested that telemedicine visits could attract reimbursement rates similar to in-person visits in academic OMFS settings and reduce indirect costs through saved travel and time, while broader adoption was perceived as contingent on supportive policies, infrastructure and co-designed pathways that address unmet needs for both patients and clinicians [1,2,5,12,16,18,27,32,33,35,36,49,51,52].

## 4. Discussion

This narrative review shows that telemedicine has been integrated into almost every major domain of OMFS, with generally favorable results for triage, routine outpatient care and postoperative follow-up, but with important caveats for high-risk oncologic and complex oral medicine patients. Across 50 studies spanning more than two decades, telemedicine has evolved from experimental teleradiology links and early video systems to routine telephone and video clinics, smartphone-based teleconsultation, structured store-and-forward networks and app-supported postoperative care [10,11,12,18,20,21,27,31,36,37,38,41,43,46,47,48,49,50,52].

### 4.1. Clinical Effectiveness and Safety

When interpreting effectiveness, safety, and concordance with in-person care, greater evidentiary weight was assigned to randomized and controlled comparative studies and to cohort studies with explicit comparators. By contrast, non-comparative service evaluations, surveys, and feasibility reports were interpreted primarily as evidence on implementation, user experience, and operational performance rather than as definitive proof of clinical equivalence.

Evidence from dentoalveolar and routine OMFS practice suggests that telemedicine can reliably support preoperative assessment and treatment planning. Early work on teleconsultations for impacted third molars and dentoalveolar surgery showed high accuracy in triaging patients to clinic versus operating room and in predicting the definitive surgical and anesthetic plan, with very low rates of cancellation or change at the time of surgery [36,37,41,44,50,52]. More recent cohorts during the COVID-19 period confirmed that most procedures planned after telemedicine consultation could be performed as intended at the subsequent in-person visit [8]. Together, these findings support the use of teleconsultation as a safe gatekeeping step for standard outpatient OMFS procedures, particularly where imaging can be reviewed remotely and where in-person examination can be reserved for cases with diagnostic uncertainty or complex comorbidity.

In trauma and emergency care, teleradiology and tele-expertise systems consistently improved triage and reduced unnecessary transfers. Hub-and-spoke networks using CT and plain radiograph transmission enabled accurate identification of surgically significant fractures and allowed many patients to be managed locally or on a scheduled basis rather than by default emergency transfer [10,28,34,42,45,48,49,50]. Prospective comparisons indicated that diagnostic performance with transmitted images was broadly acceptable, although certain fracture sites (notably frontozygomatic and infraorbital rim) remained more difficult to assess remotely, underscoring the need for high-quality imaging and clear protocols [11,47]. These data suggest that teleradiology is a robust tool for maxillofacial trauma triage, provided that its limitations are acknowledged and that thresholds for face-to-face review remain appropriately low for complex or borderline cases.

For postoperative care, randomized and controlled studies after third molar surgery found no significant differences in overall patient satisfaction between in-person and telemedicine follow-up, with perceived cost-effectiveness favoring telemedicine [9,18]. A purpose-built app (ExoDont) further demonstrated the feasibility of using mobile health tools to reinforce postoperative instructions and medication adherence, with good usability ratings [29]. These results support the substitution of a substantial proportion of routine postoperative visits with structured remote follow-up, with escalation pathways for suspected complications.

The picture is more nuanced in oncology and oral medicine. Telemedicine-based follow-up for head and neck cancer and oral potentially malignant disorders was rapidly adopted during the pandemic and was incorporated into hybrid models that combine remote and in-person visits based on risk stratification [1,12,13,14,20,24,30]. Remote imaging systems have shown that early oral squamous cell carcinoma can be detected through store-and-forward workflows from community dentists, with documented cases of early-stage diagnosis and high user satisfaction [23]. At the same time, experts in oral medicine have emphasized the limitations of telephone-only clinics for subtle mucosal disease and multisystem conditions, advocating for cautious and selective use of virtual modalities in this domain [7,15,40]. Broad regional surveys also remind us that structural barriers to early diagnosis remain significant in many low- and middle-income settings; telemedicine is perceived as part of the solution but cannot substitute for comprehensive planning, reporting, workforce and referral systems [17]. Overall, the evidence supports telemedicine as a valuable adjunct for oncologic follow-up, surveillance and early referral, but not as a full replacement for hands-on examination in high-risk or diagnostically uncertain cases.

Temporomandibular joint disorders are a further example where appropriate task-shifting is possible. A multicenter telemedicine system linking primary care with a hospital-based OMFS unit allowed most TMJ patients with myofascial pain and internal derangements to be managed conservatively in primary care, with shorter waiting times and fewer unnecessary hospital visits, while more complex arthropathies were filtered for specialist assessment [43]. This model illustrates how structured remote assessment with clear decision rules can support stratified, efficient care.

### 4.2. Patient, Clinician and System Outcomes

Across multiple settings and designs, telemedicine in OMFS has been associated with high levels of patient satisfaction, particularly for review appointments, postoperative checks, retainer reviews and benign or low-complexity conditions [2,3,7,9,11,18,19,24,28,30]. Patients valued convenience, reduced travel and time off work, and the ability to access specialist advice from home or local centers [9,11,18,28,31,32,35]. Clinician surveys similarly showed increasing acceptance of virtual clinics, especially after their enforced expansion during COVID-19, with many consultants and residents indicating that remote platforms have a permanent role in future practice [3,4,5,6,16,18,19,23,24,27,32,33,49,51]. Nevertheless, clinicians pointed to persistent concerns regarding diagnostic certainty, inability to perform a full examination, data security, medico-legal frameworks and equity of access, particularly for older or socio-economically disadvantaged patients [5,6,7,15,16,27,32,33,37,40,51].

From a health-system perspective, telemedicine has been shown to reduce unnecessary face-to-face visits, shorten time to treatment and decrease the number of avoidable transfers from peripheral to specialist centers [1,2,3,7,9,10,11,12,18,19,20,24,28,34,41,44,48,49,50]. In trauma networks, teletriage reduced the movement of patients without surgical indications, with clear savings in cost and patient discomfort [10,34,42,45,48,49,50]. In preoperative pathways, teleconsultation cut down repeated clinic visits, allowed one-stop surgery in many cases and was associated with substantial estimated savings in travel and productivity [36,37,41,44,50,52]. Financial analyses from academic OMFS practices suggest that reimbursement-to-charge ratios for telemedicine visits are broadly comparable to in-person visits, indicating that telemedicine can be economically viable for providers under contemporary reimbursement models [16]. Taken together, these findings support telemedicine not only as a clinical tool but also as a mechanism to improve system efficiency and capacity when deployed within thoughtfully designed pathways.

### 4.3. Equity, Humanitarian Work and eHealth Integration

Several studies highlight the potential of telemedicine to address geographic inequities and support humanitarian OMFS work. Tele-expertise links between European centers and partners in Africa enabled pre-mission case selection, intra-mission collaboration and postoperative follow-up, helping local teams to manage complex cases within severe resource constraints [21,22,31]. In rural or underserved regions, telehealth was feasible and acceptable for expanding access to dental and OMFS expertise, particularly where travel distances are large and local specialist availability is limited [10,20,21,28,31,32,35,39]. These experiences underline the broader role of telemedicine as an instrument of global surgery and capacity building, while also drawing attention to dependencies on infrastructure, connectivity and sustained partnerships.

Equity effects are therefore bidirectional. On the one hand, telemedicine can mitigate disparities by reducing travel burden, time off work, and geographic isolation, and by extending specialist input to rural, underserved, and humanitarian settings through hub-and-spoke or cross-border tele-expertise models [10,20,21,22,28,31,32,35,39]. On the other hand, reliance on connectivity, suitable devices, private space, and confidence in using digital tools may exacerbate inequities for older adults and socio-economically disadvantaged groups, as noted in clinician surveys and implementation reports [5,6,7,16,32,33,37,40,51]. In OMFS, an additional equity-sensitive dependency is access to imaging infrastructure: teletriage and remote decision-making often require timely radiologic review (e.g., CT/plain radiographs in trauma pathways) and adequate data transmission between peripheral sites and specialist centres [10,11,48,49,50], while store-and-forward models for oral oncology screening can depend on the availability of appropriate imaging capture in community settings and a workflow that preserves sufficient image quality for triage [23]. Where broadband, technical support, or imaging access is limited, telemedicine may unintentionally shift barriers rather than remove them. For this reason, equity-oriented implementation should include assisted-digital options (e.g., community ‘spoke’ sites that support image capture and uploading), clear hybrid pathways that preserve rapid face-to-face access when needed, and targeted patient support to improve digital and health literacy [5,6,27,32,33,39,51].

At the same time, a scoping review of eHealth in OMFS suggests that most interventions remain in feasibility or piloting phases, with limited implementation-level research and relatively few patient-centered evaluations [18]. Smartphone-based tools and low-cost networks have been shown to work well technically and educationally, and to be highly valued by trainees and students [20,27,31,35,38,46], but robust data on long-term clinical outcomes, cost-effectiveness and impact on health disparities are scarce. There is a clear need to move beyond proof-of-concept towards rigorous, patient-centered implementation studies.

### 4.4. Education, Training and Professional Practice

The rapid shift to virtual formats also affected education and training. Tele-OSCEs in OMFS were successfully implemented with performance comparable to traditional OSCEs, although both students and examiners recognized limitations in demonstrating manual skills [19]. Residency programs reported major restructuring of clinical duties, didactics and assessment, with teleconferencing and virtual teaching becoming central components of training [1,23]. Live telementoring, webinars and multi-stream operating-room feeds, as exemplified by the SEF protocol, were well received and may remain part of a “Maxillofacial Surgery 5.0” paradigm in which surgery, teaching and teleassistance are tightly integrated [20,27,31,35,38]. These developments offer opportunities for wider dissemination of expertise and more flexible education but also demand careful evaluation of learning outcomes, trainee well-being and professional identity.

### 4.5. Where Telemedicine Fits—And Where It Does Not

Synthesising across these domains, a consistent pattern emerges. Telemedicine appears particularly well-suited to:

- triage and preoperative assessment for standard outpatient OMFS procedures;
- routine postoperative follow-up and monitoring where complications are uncommon;
- maxillofacial trauma triage via teleradiology and tele-expertise;
- follow-up and survivorship care for selected oncologic and benign conditions within risk-stratified, hybrid models;
- TMJ and other conditions that can be managed conservatively with structured protocols;
- education, telementoring and multidisciplinary discussion; and
- supporting access and capacity in rural, underserved or humanitarian settings. By contrast, the evidence and expert opinion suggest that telemedicine should be used cautiously, and usually as an adjunct rather than a replacement, for:
- initial assessment of high-risk head and neck cancer and oral potentially malignant disorders;
- complex oral medicine and multisystem conditions where subtle clinical signs are critical; and
- situations where high-quality imaging or connectivity cannot be guaranteed, or where patients cannot safely use, or cannot access, digital platforms [1,7,13,14,15,17,20,30,32,37,40].

A practical but frequently under-addressed limitation is image and history collection distortion, i.e., the divergence between the patient’s true clinical status and what is captured remotely because acquisition is performed in uncontrolled environments and often with non-standardized devices [3,4,6,13,24]. In OMFS, the most consequential image-related sources of distortion are:

- Lighting and white balance (over/underexposure, mixed lighting, color shifts), which may obscure ecchymosis, erythema, mucosal color changes and subtle inflammation [3,4,38,46];
- Focus and motion blur (hand shake, low light), which reduces the visibility of lesion borders, sutures, wound dehiscence, and mucosal texture [3,4,38,46];
- Angle, perspective and working distance (foreshortening, single-view capture), which can misrepresent facial asymmetry, swelling contours, scar hypertrophy, and the apparent extent of intraoral lesions [3,4,14,38];
- Resolution and compression artifacts (messaging apps/platforms that downsample images), which degrade fine detail needed for ulcer margins, mucosal surface changes and peri-incisional assessment [10,11,12,47,48,49,50];
- Absence of scale and reference views (no ruler/landmark, no standardized poses), limiting reproducible measurements (e.g., swelling, mouth opening) and follow-up comparisons [3,4,14,38];
- Intraoral occlusion and field contamination (limited retraction, saliva/blood, fogging), which can hide posterior lesions or alter perceived surface characteristics [3,4,46].

To mitigate these issues, studies and clinical pathways should adopt standardized capture instructions (multi-angle views, consistent lighting, inclusion of a scale/reference), encourage a short video when dynamics matter (mouth opening, facial animation), prioritize platforms that preserve original file quality, and use synchronous visits for real-time coaching when needed [14,38,46]. Crucially, when image quality is insufficient or red flags emerge, protocols should mandate escalation to prompt in-person evaluation [3,4,6,53].

From a practical standpoint, specific clinical red flags and scenarios were identified in which telemedicine should not be used as a primary modality and in-person assessment should be prioritized, including:

- First presentation of suspected oral malignancy or oral potentially malignant disorders, or any new/persistent mucosal lesion where comprehensive inspection and palpation and/or biopsy planning may be required [12,13,14,15,17,23,24,30,40];
- Complex mucosal disease or multisystem oral medicine presentations in which subtle visual–tactile findings, distribution patterns, or systemic correlation are critical for diagnosis and management [4,15,40];
- Rapidly progressive facial/oral swelling, suspected deep space infection, systemic toxicity, or severe odontogenic infection, where timely examination and escalation decisions are essential [12,13,15];
- Airway or swallowing compromise (e.g., dyspnea/stridor, dysphagia, drooling) or other emergency features that mandate immediate in-person or emergency evaluation [12,13];
- New neurologic signs (e.g., paresthesia/anesthesia, cranial nerve weakness, rapidly worsening trismus) suggestive of high-risk pathology or complications requiring full clinical assessment [13,14,15];
- High-risk trauma presentations (e.g., visual disturbance, diplopia, suspected complex fractures) where diagnostic accuracy is dependent on high-quality imaging review and clinical examination, and thresholds for face-to-face assessment should remain low [10,11,47,48,49,50]; and
- Any setting in which adequate image quality, connectivity, privacy, or safe patient use of digital tools cannot be ensured, as this may amplify diagnostic uncertainty and inequity [1,7,13,14,15,17,20,30,32,37,40].

These boundaries will likely shift as technology, remote examination techniques and regulatory frameworks evolve, but clear local criteria for what is “telemedicine-appropriate” remain essential.

### 4.6. Strengths and Limitations of the Evidence and of This Review

This review has several strengths. Multiple major biomedical and technical databases were searched on a defined date using broad, OMFS-specific telemedicine terms, and study selection and data extraction were performed in duplicate with adjudication by a third reviewer, which should reduce selection bias. The inclusion of older foundational work alongside pandemic-era and post-pandemic studies provides a long-term perspective on how telemedicine has matured within OMFS [10,11,41,45,46,47,48,49,50,51,52]. The synthesis spans clinical outcomes, patient and clinician experiences, organizational models and humanitarian applications, offering a broad, specialty-specific view.

However, important limitations must be acknowledged. First, the underlying evidence base is heterogeneous and often methodologically limited. Many studies are single-center, observational, descriptive or pilot in nature, with small sample sizes and short follow-up, and few apply standardized outcome measures or formal economic evaluation [8,9,10,11,12,18,20,27,28,34,36,37,41,42,43,44,48,49,50]. Notably, well-powered multicenter randomized controlled trials directly comparing telemedicine with standard in-person consultations, as well as rigorous cost-effectiveness or cost-utility analyses, remain scarce; consequently, definitive conclusions on equivalence or superiority across OMFS indications cannot yet be drawn. Publication bias towards positive experiences is likely. Second, although this review used a structured search and explicit eligibility criteria, it was not registered as a systematic review and did not apply formal risk-of-bias tools uniformly across all study types. Third, only English-language studies were included, and telemedicine experiences reported in other languages or in grey literature may have been missed. Finally, the rapid evolution of telehealth policy and technology means that some early findings, especially regarding connectivity and reimbursement, may be less applicable to current practice.

Conversely, ongoing advances in digital infrastructure, secure platforms, remote examination and imaging technologies, together with increasingly mature regulatory and reimbursement frameworks, may progressively reduce current implementation barriers and facilitate larger pragmatic trials and comprehensive economic evaluations.

### 4.7. Implications for Practice and Research

For clinical practice, the available evidence supports a deliberate, pathway-based integration of telemedicine into OMFS services. Routine follow-up and selected preoperative assessments can be safely and acceptably delivered remotely when supported by clear triage criteria, access to imaging and robust escalation mechanisms. Trauma networks and oncology services should consider formalizing teleradiology and hybrid follow-up models, respectively, while ensuring that in-person assessment remains accessible for high-risk or complex patients. Humanitarian and rural programs can leverage tele-expertise and smartphone-based tools to extend specialist input, but should plan for long-term infrastructural and training needs. Equity-sensitive evaluation should be incorporated into these pathways by reporting access-related metrics (e.g., mode of visit, failed connections, need for assisted imaging, and uptake by older or disadvantaged patients) and by explicitly addressing digital-divide barriers, technological literacy, and access to imaging and secure platforms during service design [5,6,16,27,32,33,37,40,51].

For research, priorities include: high-quality comparative studies of telemedicine versus in-person care across key OMFS indications; standardized patient-reported outcome measures; detailed economic and equity analyses; and implementation science approaches that examine context, adoption and sustainability. Patient and clinician co-design should be embedded in the development of telemedicine pathways, especially for vulnerable populations. As eHealth interventions become more sophisticated, AI-enabled components may play a specific role in (i) automated image quality assurance (e.g., detection of blur, underexposure, insufficient field-of-view, or compression-related loss of detail) [53,54,55,56], (ii) triage support and risk stratification by combining structured remote histories/checklists with image and radiology inputs [57,58,59,60,61], and (iii) decision support in high-volume pathways such as trauma teleradiology and image-based oral cancer triage. In principle, such tools could reduce avoidable escalation while improving safety by systematically flagging low-confidence cases for prompt face-to-face assessment [62,63,64,65]; however, prospective validation in OMFS-specific settings, transparency of performance, bias assessment, and clear governance for data protection and liability will be required prior to implementation at scale.

Overall, telemedicine in OMFS has moved from niche innovation to an essential component of modern practice. The challenge now is to consolidate the gains made during the pandemic into evidence-based, equitable and patient-centred models of care that recognize both the power and the limits of remote surgery-adjacent medicine.

## 5. Conclusions

In conclusion, telemedicine has transitioned from experimental pilots to a core, though carefully circumscribed, component of OMFS practice, with the greatest maturity in dentoalveolar care, trauma triage, routine follow-up and education. Across 50 heterogeneous studies, evidence from comparative designs in selected settings suggests high concordance with in-person decision-making and favorable workflow and satisfaction outcomes, particularly when telemedicine is embedded in structured, risk-stratified pathways; however, much of the remaining literature is observational or feasibility-oriented, and broad claims of equivalence to in-person care across OMFS indications should therefore be made cautiously. At the same time, the evidence reinforces that telemedicine should complement—not replace—hands-on assessment in high-risk head and neck oncology, complex oral medicine and diagnostically uncertain presentations, where subtle clinical signs and comprehensive examination remain indispensable.

Telemedicine also holds promise for reducing geographic inequities and strengthening humanitarian and rural programs, provided that infrastructural, regulatory and digital-divide barriers are actively addressed. Moving forward, the consolidation of telemedicine in OMFS will depend on robust comparative and economic studies, standardized outcome measures, and implementation strategies co-designed with patients, clinicians and health systems. The integration of AI-enabled imaging, decision support and remote monitoring offers additional opportunities but will require clear specialty-specific standards, governance and medico-legal frameworks. If these conditions are met, telemedicine is poised to remain a stable, equitable and patient-centred pillar of OMFS care in the post-pandemic era.

## Figures and Tables

**Table 1:** Evidence map of telemedicine in OMFS.

Clinical Domain	Typical Use-Cases	Predominant Telemedicine Modalities	Comparative Evidence Present? *	Key Recurring Patterns/Trends
Implementation/attitudes/economics [2,3,4,5,6,7,9,11,15,16,18,19,27,28,32,33,37,40,49,51]	Adoption; barriers/facilitators; medico-legal/privacy concerns; satisfaction; service redesign; reimbursement	Mainly telephone/video; mixed remote models; workflow guidance	Limited	High acceptability for selected indications; persistent concerns about examination limits, confidentiality and governance; strong dependency on infrastructure and pathway design
Routine outpatient care & preoperative triage [1,2,3,7,8,9,11,12,14,18,19,22,24,32,36,37,40,41,42,44,50,52]	New patient triage; preoperative assessment; outpatient review/virtual clinics; treatment planning	Telephone/video clinics; hybrid models; selective store-and-forward	Yes (subset)	High plan/concordance reported in low-risk settings; reduction of unnecessary face-to-face visits; escalation needed when diagnostic uncertainty exists
Postoperative follow-up & mHealth-supported monitoring [9,29]	Postoperative review (e.g., third molars); adherence support and reminders	Video/tele-visit; app-based tools	Yes (limited)	Satisfaction comparable in selected settings; convenience and perceived cost-effectiveness; app-based approaches feasible but evidence remains early
Trauma & emergency care (teleradiology/tele-expertise) [10,11,34,39,42,45,47,48,49,50]	Trauma referral triage; remote imaging review; transfer appropriateness	Teleradiology systems; tele-expertise/videoconferencing	Yes (subset)	Improved triage and fewer avoidable transfers; image transmission quality and timeliness are pivotal; some fracture sites remain challenging remotely
Humanitarian/equity/access models [10,20,21,22,28,31,32,35,39]	Mission planning; remote specialist support for underserved settings; hub-and-spoke access models	Teleconsult links; online collaboration tools; hybrid models	Mostly no	Feasibility and perceived value for extending specialist input; dependency on connectivity, training, and sustainable partnerships
Oncology/oral cancer (follow-up & early detection/triage) [1,12,13,14,17,20,22,23,24,30]	Risk-stratified follow-up; early detection/triage using images; OPMD/oral cancer pathways	Hybrid phone/video; asynchronous image-based workflows	Limited	Useful in structured follow-up; potential triage role; caution for first presentation/new lesions and diagnostically uncertain mucosal disease
Oral medicine/complex mucosal disease [7,15,37,40]	Counselling and follow-up; chronic/complex mucosal conditions	Mainly telephone/remote clinics	No	Helpful for continuity and counselling; limitations for nuanced diagnosis where detailed visual–tactile examination is critical
Education & training/telementoring [19,20,33]	Tele-OSCEs; remote teaching; residency program adaptation; telementoring	Video platforms; streaming; structured teleteaching	Yes (limited)	Feasible for communication/knowledge components; limitations remain for hands-on technical skill assessment
TMJ/TMD pathways and TMJ-related tele-support [43,47]	TMD management support from primary care; TMJ-related procedural/tele-support experiences	Store-and-forward networks; interactive teleconsult/video support	Yes (limited)	Suggests efficiency gains in selected pathways; broader generalization limited by heterogeneity

* Comparative evidence refers to randomized, quasi-experimental, or diagnostic-accuracy comparisons; it remains a minority of the overall evidence base.

## Data Availability

No new data were created or analyzed in this study. Data sharing is therefore not applicable to this article. All data supporting the findings of this narrative review are contained within the article itself and in the publicly available studies cited in the reference list.

## Supplementary Materials

The following supporting information can be downloaded at: https://www.mdpi.com/article/10.3390/jcm15020452/s1, Table S1: Full database search strategies; Table S2: Characteristics of the 50 studies included in the narrative review.
