# Supplementary material for: Telemedicine in Oral and Maxillofacial Surgery: A Narrative Review of Clinical Applications, Outcomes and Future Directions
Source: J Clin Med. 2026 Jan 7;15(2):452. doi: 10.3390/jcm15020452 (PMC12842303; doi:10.3390/jcm15020452)
Supplement: Supplementary file 1 [file jcm-15-00452-s001.zip › jcm-4058646-supplementary.pdf]

Supplementary Table S1. Full database search strategies

Database	Exact Search String
PubMed/MEDLINE	((Telemedicine[Mesh] OR "telemedicine"[tiab] OR "telehealth"[tiab] OR "tele-health"[tiab] OR "teleconsult*"[tiab] OR "tele-consult*"[tiab] OR "videoconferenc*"[tiab] OR "video consult*"[tiab] OR "video visit*"[tiab] OR "virtual clinic*"[tiab] OR "teleradiolog*"[tiab] OR "teledent*"[tiab] OR ("remote"[tiab] AND (consult*[tiab] OR monitor*[tiab] OR imaging[tiab] OR assessment[tiab])) OR "store-and-forward"[tiab])) AND (("Oral and Maxillofacial Surgery"[Mesh] OR "Maxillofacial Injuries"[Mesh] OR "Dentistry, Operative"[Mesh] OR "Oral Surgery"[tiab] OR "oral and maxillofacial surg*"[tiab] OR "OMFS"[tiab] OR "maxillofacial"[tiab] OR "dentoalveolar"[tiab] OR "third molar*"[tiab] OR "wisdom tooth*"[tiab] OR "temporomandibular"[tiab] OR "TMJ"[tiab] OR "oral cancer"[tiab] OR "head and neck"[tiab] OR "orthodontic*"[tiab] OR "oral medicine"[tiab]))) NOT (Editorial[pt] OR Comment[pt] OR Letter[pt] OR Case Reports[pt]) Filters: English, Humans
Embase (Elsevier, embase.com)	'telemedicine'/exp OR telehealth:ti,ab OR 'tele consultation':ti,ab OR teleconsult*:ti,ab OR videoconferenc*:ti,ab OR 'virtual clinic*':ti,ab OR 'teleradiology'/exp OR teleradiolog*:ti,ab OR teledent*:ti,ab OR ('remote':ti,ab AND (consult*:ti,ab OR monitor*:ti,ab OR imaging:ti,ab OR assessment:ti,ab)) OR 'store and forward':ti,ab AND ('maxillofacial surgery'/exp OR 'oral surgery':ti,ab OR 'oral and maxillofacial':ti,ab OR omfs:ti,ab OR maxillofacial:ti,ab OR dentoalveolar:ti,ab OR 'third molar':ti,ab OR 'wisdom tooth':ti,ab OR 'temporomandibular joint'/exp OR temporomandibular:ti,ab OR tmj:ti,ab OR 'oral cancer'/exp OR 'head and neck':ti,ab OR orthodontic*:ti,ab OR 'oral medicine':ti,ab) AND [english]/lim AND [human]/lim NOT ([editorial]/lim OR [letter]/lim OR [note]/lim OR 'case report'/exp)
Scopus (Elsevier)	TITLE-ABS-KEY (telemedicine OR telehealth OR "tele consultation" OR teleconsult* OR videoconferenc* OR "virtual clinic*" OR teleradiolog* OR teledent* OR ("remote" W/3 (consult* OR monitor* OR imaging OR assessment)) OR "store-and-forward") AND TITLE-ABS-KEY ("oral and maxillofacial" OR omfs OR maxillofacial OR "oral surgery" OR dentoalveolar OR "third molar*" OR "wisdom tooth*" OR temporomandibular OR tmj OR "oral cancer" OR "head and neck" OR orthodontic* OR "oral medicine") AND (LIMIT-TO (LANGUAGE , "English")) AND NOT (DOCTYPE (ed OR le OR no))
Web of Science Core Collection	TS=(telemedicine OR telehealth OR teleconsult* OR "tele consultation" OR videoconferenc* OR "virtual clinic*" OR teleradiolog* OR teledent* OR ("remote" NEAR/3 (consult* OR monitor* OR imaging OR assessment)) OR "store-and-forward") AND TS=("oral and maxillofacial"

Database	Exact Search String
	OR OMFS OR maxillofacial OR "oral surgery" OR dentoalveolar OR "third molar*" OR "wisdom tooth*" OR temporomandibular OR TMJ OR "oral cancer" OR "head and neck" OR orthodontic* OR "oral medicine") Refined by: LANGUAGES=(ENGLISH) AND DOCUMENT TYPES=(ARTICLE OR REVIEW OR PROCEEDINGS PAPER) Exclude: DOCUMENT TYPES=(EDITORIAL MATERIAL OR LETTER)
Cochrane Library (CENTRAL & Reviews)	([mh "Telemedicine"] OR telemedicine:ti,ab,kw OR telehealth:ti,ab,kw OR teleconsult*:ti,ab,kw OR videoconferenc*:ti,ab,kw OR teleradiolog*:ti,ab,kw OR teledent*:ti,ab,kw OR ("remote" NEXT (consult* OR monitor* OR imaging OR assessment)):ti,ab,kw OR "store and forward":ti,ab,kw) AND ([mh "Oral and Maxillofacial Surgery"] OR "oral and maxillofacial":ti,ab,kw OR OMFS:ti,ab,kw OR maxillofacial:ti,ab,kw OR "oral surgery":ti,ab,kw OR dentoalveolar:ti,ab,kw OR "third molar*":ti,ab,kw OR temporomandibular:ti,ab,kw OR TMJ:ti,ab,kw OR "oral cancer":ti,ab,kw OR "head and neck":ti,ab,kw OR orthodontic*:ti,ab,kw OR "oral medicine":ti,ab,kw)
IEEE Xplore	(("telemedicine" OR "telehealth" OR "teleconsult*" OR "videoconference*" OR "teleradiology" OR "teledent*" OR ("remote" NEAR/3 (consult* OR monitoring OR imaging OR assessment)) OR "store-and-forward") AND ("oral and maxillofacial" OR "maxillofacial" OR "oral surgery" OR "dentoalveolar" OR "third molar" OR "temporomandibular" OR "TMJ" OR "oral cancer" OR "head and neck" OR "orthodontic" OR "oral medicine")) Filters: Publication Year = All; Content Type = Journals & Conferences; Language = English

Supplementary Table S2. Characteristics of the 50 studies included in the narrative review

No.	Author	Year	Country/Setting	Study Design	Modality	Indication/Use Case	N (sample size)	Key Outcomes
1	Kaibuchi et al. [23]	2025	Japan; local dental clinics; 2017–2022	Case series + pilot (service program evaluation)	Asynchronous remote imaging via mail-form; email reports	Early detection of oral cancer; diagnostic support	38 clinics; 114 consultations; 49M/65F	Provisional vs actual diagnosis concordance 53.8%; malignancy suspected in 13, confirmed in 3 (23.1% of suspects: 2 tongue, 1 buccal mucosa); 71.4% dentists 'very satisfied'
2	Hans et al. [3]	2025	UK; single acute hospital trust; Mar–Dec 2020	Retrospective service evaluation	Remote appointments (phone/video; not specified per mode)	Orthodontics (retainer reviews) and OMFS new patient triage/follow-up	901 remote appts (387 Ortho; 523 OMFS)	FTA low (OMFS 3%, Ortho 6%); Ortho: 83% retainer reviews; Discharge after attendance: Ortho 49%; OMFS new patient remote 41%; Saved F2F: Ortho 92%, OMFS 81%
3	Hans et al. [4]	2025	UK; 6 NHS hospital trusts	Cross-sectional questionnaire (clinicians)	Remote appointments (general)	Orthodontics & OMFS clinician perceptions	36 responses; 28 completed (21 orthodontic; 7 OMFS)	High clinician satisfaction with clinician confidentiality; concerns about patient confidentiality and inability to examine; 75% felt remote has a place post-pandemic (retainer reviews, biopsy results)
4	Grillo et al. [22]	2025	Angola mission + Brazil collaborators; Luanda (Mar 2023)	Mixed: pre-mission online survey + observational mission report	Internet-based tools for planning/collaboration/education	Humanitarian maxillofacial surgery mission planning and execution	Survey (numbers NR in abstract); treated 26 emergency cases + 17 inpatients	Identified resource shortages; complex surgeries performed; online tools improved planning/execution; educational sessions delivered
5	Masongo et al. [5]	2024	Australia; consultants nationwide	Mixed-methods (survey + interviews)	Telehealth services (general)	OMFS consultants' barriers and willingness post-COVID	42 respondents	82% willing to continue telehealth; more experience (≥2–3 years) associated with willingness (p=0.028); themes: access, addressing needs, diagnostic uncertainty, limits as postop observation tool

6	Welham et al. [6]	2024	Australia; John Hunter Hospital (NSW)	Commentary/review with local outpatient data (2020–2023)	Audio/video telehealth in outpatients	OMFS outpatient care during COVID-19	NR in abstract	Significant uptake during COVID-19; ethical/legal considerations under-analyzed; need system improvements before sufficiency/adequacy
7	Martinez-Ramirez et al. [17]	2024	Latin America & Caribbean; 21 countries	Cross-sectional online survey	Telemedicine and online education (perceptions)	Barriers to early diagnosis/management of oral cancer/OPMD	23 professionals	Major barriers: limited plans, reporting, referral pathways, shortage of trained professionals; endorsed utility of online education (100%) and telemedicine (91.3%)
8	Deol et al. [27]	2024	USA	Narrative literature review	Telemedicine in OMFS (general)	Access disparities; rural populations	NA (review)	Advocates broader adoption; potential to improve access, reduce travel, support collaboration; contingent on policy/infrastructure
9	Miranda-Hoover et al. [2]	2024	USA; university health system; 2019–2022	Retrospective EMR analysis + provider survey	Telehealth (video/phone; unspecified split)	OM & OMFS specialty services	OMFS telehealth use 12%; OM 8%; majority (87%) return patients	Telehealth decreased in year 2 (p=0.0001); by Aug 2022, NP telehealth 0–1.5%, RP telehealth ~11.4%; providers rate telehealth effective complement (4.2/5)
10	Seifert et al. [19]	2022	Germany; Frankfurt	Pilot comparative study (Tele-OSCE vs prior in-person OSCE)	Tele-OSCE via Zoom	Dental/OMFS education and assessment	66 students (34 tele-OSCE; 32 prior in-person); 9 examiners	No significant difference in performance (p=0.53); limitation in demonstrating practical skills; anamnestic/consultation competencies feasible
11	McIntosh et al. [28]	2022	UK; Humberside OMFS unit	Survey (patients + clinicians) of rapid telephone review service	Telephone consultations	Maintain access during cessation of routine care	199 patients contacted; 93 analyzable patient surveys; 7/12 staff responses	High satisfaction; 37% patients discharged via telephone review; suggestions include new patient use, webcam addition
12	Gangwani et al. [8]	2023	USA; University of Rochester/EIOH; Mar 2020–Mar 2021	Retrospective cohort	Telemedicine pre-op consultations	Planned OMS procedures (mostly dentoalveolar)	443 telemedicine consults (97.3% dentoalveolar)	98.19% treated at following appointment; age/gender not associated with plan change
13	Krishna et al. [29]	2021	India; academic + software team	Prototype development & testing (uMARS)	Android app (ExoDont) for postop reminders/medication adherence	Post-extraction postoperative care	uMARS ratings; specific user N not reported in abstract	Highest score: Perceived Impact (mean 4.6); lowest: Engagement (3.5)
14	Kanatas et al. [30]	2023	UK; Aintree and Leeds HNC centers	Service review of follow-up patterns	Telephone/video + face-to-face hybrid	Head & neck cancer follow-up	Alive at lockdown start: 212; post-lockdown data across 5–7 months	First post-lockdown consultations: Aintree 67% phone/33% F2F; Leeds 78% phone/22% F2F; second consultations

								shifted towards more F2F in Aintree
**15**	Nadella et al. [16]	2021	USA; University of Pennsylvania OMS practice	Retrospective cohort (billing claims)	Telemedicine vs in-person office visits	Financial reimbursement comparison	6,082 claims; 4,045 patients	Mean reimbursement: tele $98.07 (RC 0.48) vs in-person $109.5 (RC 0.50); overall RC ratio diff 2% (p=0.001); no significant diff when stratified by new/established
**16**	Macken et al. [15]	2021	UK; Oral Medicine unit, London	Reflective piece	Remote telephone clinics	Oral Medicine and Behçet's disease management	NA (reflection)	Virtual clinics useful but insufficient for this specialty; highlights limits of visual assessment reliance
**17**	Robiony et al. [14]	2021	Italy; Academic Hospital of Udine	Organizational model description (experience report)	Telemedicine/video consultation; 'telesemiology'	Reorganization during COVID-19; outpatient care and follow-up	78 patients via teleconsultation (Mar–Apr 2020)	Telemedicine central to continuity; defined signs/symptoms for remote assessment; prioritized oncology follow-up
**18**	Champion et al. [9]	2021	USA; Thomas Jefferson University	Randomized comparison (telemedicine vs in-person)	Telemedicine postoperative appointment	Postoperative review after third molar surgery	69 randomized; 47 completed (24 in-person; 23 telemedicine)	No difference in overall satisfaction (46.46 vs 48.78/50; p=0.11); higher perceived cost-effectiveness in telemedicine (p=0.01)
**19**	Horgan et al. [7]	2021	UK; Shrewsbury and Telford	Retrospective patient survey	Telephone consultations	OMFS pandemic-era consultations	Records reviewed 150; 135 included; 109 responses (80.7%)	High satisfaction (G-MISS); 83.48% would accept future phone consult; lower compliance in new vs review patients (p=0.004)
**20**	Barca et al. [12]	2020	Italy; Catanzaro University hospital	Prospective service model report	Video/phone messaging (WhatsApp/Telegram) + questionnaires	Oncologic follow-up, chronic lesions (A1/A2); first urgent visits (B1/B2 incl. MRONJ, abscess, TMJ dislocation)	90 patients	High satisfaction among patients and doctors; improved accessibility; reduced COVID-19 spread risk (per authors)
**21**	Singh et al. [31]	2020	India (literature review context)	Narrative review of teleassistance/teleconsultation using smartphones	Smartphone-enabled teleassistance/teleconsultation	Specialist consultation, diagnosis, treatment, follow-up in remote areas	15 articles reviewed (per abstract)	Concludes smartphone teleconsultation is useful for remote specialized support
**22**	Moon et al. [32]	2021	USA (University of Pennsylvania)	Best-practice guidance article	Telemedicine encounter optimization	OMFS telemedicine during COVID-19	NA	Presents best practices to optimize OMFS telemedicine encounters
**23**	Brar et al. [33]	2021	USA (OMFS programs nationwide)	Program director survey (cross-sectional)	Telemedicine and modified in-person triage protocols	Impact on OMFS training programs early pandemic	95 programs approached; 33 responses (35%)	100% suspended electives; telemedicine used by 40%, modified in-person 51%; PPE shortage 51%; preferred PPE N95/full face shield (63%); 21% PAPRs; resident wellness

								resources 73%; virtual didactics common
**24**	Robiony et al. [13]	2020	Italy; University Hospital of Udine	Organizational model description	Teleconsultation; virtual rooms; 'telesemiology' checklist	Outpatient OMFS services during lockdown	NR in abstract	Model translating consultations to virtual environment; clinical examples; introduces telesemiology and checklist
**25**	Al-Izzi et al. [24]	2020	UK; large teaching hospital	Clinician survey + prospective virtual clinic outcomes	Virtual consultations/telemedicine	Pandemic-era virtual clinics acceptance and outcomes	151 consultations analyzed; survey of consultants (N not specified)	149/151 (98.7%) achieved working diagnosis/treatment plan or concluded care without conversion; overall consultations reduced vs previous year (1,223 vs 465)
**26**	Chiguru pati et al. [1]	2020	USA (multi-institution authorship)	Expert review/position paper	Telemedicine and workflow changes	COVID-19 era considerations for OMFS	NA	Recommends embracing telemedicine alongside infection control, screening, and workflow redesign
**27**	Tel et al. [20]	2020	Italy; Academic Hospital of Udine	Protocol description + educational evaluation	SEF telemedicine protocol (Smart videosurgery, Easy teleteaching, Fast teleassistance)	Telementoring, distance surgery visualization, webinars	9 operations; 90 students (10 per procedure)	Interface stable/high quality; student satisfaction index 9/10
**28**	Brucoli et al. [10]	2019	Italy; Piedmont hub center (Novara)	Retrospective service review	Teleradiology (Tempore system)	Triage of maxillofacial trauma referrals	467 patients; 605 fractures (2014–2018)	Surgery suggested in 68 after remote CT; 223 no surgery; 176 needed clinical assessment—afterward only 27 had surgery
**29**	Ambroise et al. [34]	2019	France; Normandy region	Implementation report (preliminary results)	Mobile tele-expertise app (Therap-e platform)	Emergency department tele-expertise for maxillofacial trauma and conditions	NR in abstract	Secure transmission and documentation of photos/consults; addresses demographic and quality/safety challenges
**30**	Van den Bosch et al. [18]	2019	Netherlands (Radboud UMC)	Scoping review	Patient-centred eHealth (incl. video-teleconsultation)	OMFS eHealth interventions mapping	41 papers; 34 unique interventions	19 for head & neck cancer; 11 video-teleconsultation; most studies feasibility/piloting (26), 8 evaluation, 7 development; no implementation studies
**31**	Ambroise et al. [21]	2018	France–Mali telelink	Experience report	Teleconsultations between Caen (France) and Bamako (Mali)	Humanitarian mission prep and postoperative follow-up	4 teleconsultations; 21 patients assessed	Enabled preselection, imaging review, postoperative follow-up; facilitated therapeutic decisions and planning
**32**	Martin et al. [35]	2016	USA (South Carolina)	Survey of dentists (SC Dental Association)	Teledentistry interest/knowledge	Access to care improvements for underserved	N not stated in abstract (members surveyed); analyses	69.3% had some/no teledentistry knowledge; identified consult needs (endodontics 40.2%,

							reported by percentages	OMFS 37.9%, etc.); higher Medicaid share associated with more intended uses
**33**	Wood et al. [36]	2016	USA (Virginia)	Dual survey (GPs and OMSs)	Telemedicine consultations (perceptions and demand)	Access to care; consultation efficiency	226 general practitioners; 41 OMS respondents	Rural vs urban differences (distance, referral volume); GPs would refer more if tele-consults available; OMSs influenced by increased referrals
**34**	Wood et al. [37]	2016	USA (Virginia Commonwealth University)	Retrospective 6-year follow-up study	Telemedicine consultations (store-and-forward?)	Preoperative assessment and triage	335 patients	Triage correct 99.6%; sufficient assessment 98.0%; accurate diagnosis/treatment plan 95.9%; estimated cost savings $134,640
**35**	Carey et al. [38]	2015	UK; trainees in OMFS	Survey of trainees	Smartphone and medical app use	Productivity, decision-making, telemedicine phenomenon	NR in abstract (percentages reported)	94% own smartphone; 61% iPhone; 89% downloaded medical apps and use regularly
**36**	Mladenović et al. [39]	2013	Serbia; Faculty of Medicine Nis	Case reports (3 cases)	Telemedicine consulting via XPA3 Online; smartphone/computer	Preparation and planning of prosthetic tooth replacement	3 cases	All consultations successful; no need for further regional center procedures
**37**	Blomstrand et al. [40]	2012	Sweden (Uppsala University Hospital); EU Health Optimum project	Service description with early outcomes	Secure interactive telemedicine rounds/consultations	Oral medicine/OMFS consultations and referrals	10 patients discussed in rounds	2 referred to specialist; 8 managed with diagnosis/treatment suggested without referral
**38**	Brockes et al. [41]	2012	Switzerland; University Hospital Zurich	Content analysis of online consultation service	Medical online consultation (since 1999)	Queries regarding maxillofacial surgery	204 questions evaluated	Users: mean age 38; topics included sinusitis (21), aphthae (17), BCC (14); provided information/advice patterns
**39**	Hutchison et al. [42]	2012	Scotland; Aberdeen/Grampian region	Retrospective audit	Telemedicine referral system usage	Adult facial trauma referrals	137 patients identified	Only 1 referral used telemedicine; indicates underuse
**40**	Salazar-Fernandez et al. [43]	2012	Spain; Seville region	Quasi-experimental multicenter comparison (tele vs conventional)	Store-and-forward telemedicine system (SFTMS)	TMJ disorder management from primary care	Hospital conventional: 710; Teleconsultation: 342	High-resolution: 89.7% managed non-surgically in primary care; time to treatment 2.3 days tele vs 78.6 days conventional ($p<0.05$); lost working hours 16 vs 32 ($p<0.05$)
**41**	Herce et al. [44]	2011	Spain; Seville	Longitudinal descriptive pilot	Store-and-forward telemedicine (SFTMS)	Presurgical management of impacted third molars	97 patients; 102 teleconsultations	Mean wait to surgical list 3.33 days tele vs 28 days conventional; similar cancellation rates (7.8% vs 8.85%)
**42**	Truppe et al. [45]	2011	Austria (biotelematics institute)	Perspective/conceptual article	Teleconsultation in craniomaxillofacial surgery	Concepts and future perspectives	NA	Discusses potential and perspectives; no empirical results

**43**	Aziz et al. [46]	2009	USA; academic setting	Descriptive viewpoint	Smartphone-based telemedicine for consultation/communication	Specialist consultation & triage	NA	Argues for efficiency/effectiveness of smartphone telemedicine for OMFS
**44**	Ewers et al. [47]	2005	Austria; Vienna	Seven-year clinical experience report	Interactive teleconsultation; ISDN/TCP-IP; UMTS video streaming	Range of CMF procedures incl. TMJ arthroscopy, orthognathic planning	50 telemedically supported treatments; 60 UMTS video streamings	All intraoperative applications completed; UMTS: 48/60 uninterrupted, 8 slight interruptions, 4 breakdowns; sufficient resolution to diagnose small TMJ structures
**45**	Roccia et al [48]	2005	Italy; Turin (PATATRAC system)	Two-year clinical experience	Telemedicine system (PATATRAC)	Management of maxillofacial trauma referrals	18 tele-consultations from 35 hospitals	Only 50% required maxillofacial treatment; only 1 immediate transfer; reduced unnecessary transfers
**46**	Brownrigg et al. [49]	2004	UK; Federation model across 4 hospitals	Descriptive account	Videoconferencing telemedicine for OMFS trauma referrals	Improve appropriateness of transfers; develop ER skills	NR in abstract	Increased appropriateness of transfers; believed improved early management
**47**	Jacobs et al [11]	2002	UK; Manchester	Prospective diagnostic accuracy study	Telemedicine system vs plain radiography	Diagnosis of facial fractures	20 radiographs (10 with, 10 without fractures); 8 OMFS + 8 A&E doctors	Fracture position more accurate with plain radiography; OMFS via telemedicine broadly comparable to A&E with plain films; poorer for frontozygomatic/infraorbital rim via telemedicine
**48**	Lowry et al. [50]	2001	UK; four towns (NW England)	Service development report (first 12 months)	Videoconferencing across A&E departments to central OMFS unit	Remote consultations and radiograph visualization	Average 25 remote consultations/month (12 months)	Favourable feedback from patients and clinicians
**49**	Coulthard et al. [51]	2000	UK; Greater Manchester GDPs	Postal questionnaire	Telemedicine interest in oral surgery referrals	Referral system evaluation and demand for telemedicine	400 mailed; 84% participation	48% dissatisfied with current referral site; distance a concern; 23% want better communication with surgeon; 70% want involvement in consultation; demand for telemedicine inferred
**50**	Rollert et al. [52]	1999	USA; Medical College of Virginia	Retrospective telemedicine consultation evaluation	Telemedicine preoperative assessment	Dentoalveolar surgery under GA with nasotracheal intubation	43 telemedicine consults; 35 treated	95% underwent surgery at immediate appointment; 100% correctly assessed; 2 ASA III required further evaluation; no cancellations or anesthetic complications attributable to telemedicine assessment
